# ﻿Four species of *Zoica* Simon, 1898 from Asia (Araneae, Lycosidae)

**DOI:** 10.3897/zookeys.1249.152933

**Published:** 2025-08-25

**Authors:** Jin-Zhen Lu, Yuri M. Marusik, Zhi-Sheng Zhang, Lu-Yu Wang

**Affiliations:** 1 Key Laboratory of Eco-environments in Three Gorges Reservoir Region (Ministry of Education), School of Life Science, Southwest University, Chongqing 400715, China Southwest University Chongqing China; 2 Institute for Biological Problems of the North RAS, Portovaya Str. 18, Magadan 685000, Russia Institute for Biological Problems of the North RAS Magadan Russia; 3 Department of Zoology & Entomology, University of the Free State, Bloemfontein 9300, South Africa University of the Free State Bloemfontein South Africa; 4 Altai State University, Lenina Pr., 61, Barnaul, RF-656049, Russia Altai State University Barnaul Russia

**Keywords:** Bhutan, China, new record, taxonomy, Thailand, webs, wolf spider, Zoicinae

## Abstract

Three new species of the wolf spider genus *Zoica* Simon, 1898 are described from eastern and southeastern parts of Asia: *Zoica dulong* Lu, Zhang & Wang, **sp. nov.** (Yunnan, ♂♀) and *Z. medogensis* Lu, Zhang & Wang, **sp. nov.** (Xizang, ♂♀) from China and *Z. thailandica* Lu, Zhang & Wang, **sp. nov.** (Ratchaburi, ♂♀) from Thailand. The male of *Z. oculata* Buchar, 1997 (Xizang) is described here for the first time, and the first record of the species from China is reported. Descriptions and photographs of all the species are provided.

## ﻿Introduction

Spiders of the genus *Zoica* Simon, 1898, are among the smallest within the family, measuring only 0.99 to 4.26 mm in body length (Wang et al. 2021). Currently, the genus contains twelve species distributed in northern Australasia, southern China, India and Southeast Asia, out of which four species have been reported based on females only (WSC 2025). Although *Zoica* was considered in several publications, it was never the subject of a global revision.

While studying lycosids in China and adjacent regions, we recognized three new species and a previously unknown male of *Z. oculata* from Bhutan. Furthermore, we report the presence of webs in *Zoica* spiders for the first time.

## ﻿Material and methods

All specimens were preserved in 75% ethanol. The specimens were examined, measured, and photographed using a Leica M205A stereomicroscope equipped with a Leica DFC450 camera, and LAS software (ver. 4.6). The left male palp was used for photography. The epigyne was cleared by immersing it in pancreatin solution before examination and photography (Álvarez-Padilla and Hormiga 2007). Leg measurements are shown as total length (femur, patella and tibia, metatarsus, tarsus). All measurements are in millimeters. All specimens examined here are deposited in the spider collection at the School of Life Sciences, Southwest University, Chongqing, China (SWUC). Terminology as follows Li et al. (2013), Framenau et al. (2009) and Sierwald (2000).

Abbreviations used in the text and figures: ALE–anterior lateral eye; AME–anterior median eye; PLE–posterior lateral eye; PME–posterior median eye.

## ﻿Taxonomy

### ﻿Family Lycosidae Sundevall, 1833 (狼蛛科)

**Subfamily Zoicinae Lehtinen & Hippa, 1979** (佐卡蛛亚科)

#### ﻿Genus Zoica

Taxon classificationAnimaliaAraneaeLycosidae

Simon, 1898

CC0E3398-0284-5BD4-A323-B1E950FA176F

##### Type species.

*Zobia parvula* Thorell, 1895

##### Diagnosis.

*Zoica* can be diagnosed by the following characteristics: male opisthosoma with dorsal scutum; the gently sloping margins of the cephalic area; articulated tegular apophysis absent; lateral apophysis present; embolus thin, curved spine-like, positioned apically on the bulb and mostly covered by tegulum in ventral view; epigynum variable, often protruding scape-like posteriorly (Framenau et al. 2009).

##### Composition and distribution.

Twelve species are known from Australasia, southern China, India and Southeast Asia.

#### ﻿ Zoica dulong

Taxon classificationAnimaliaAraneaeLycosidae

Lu, Zhang & Wang sp. nov.

35085F67-6A94-5D71-830F-360E03448E2D

https://zoobank.org/2833B060-4346-4BAC-8F83-FC9C99E7BAAB

Figs 2 , 3 , 10 独龙佐卡蛛

##### Type material.

***Holotype*** • ♂ (SWUC-T-LY-28-01), China, Yunnan Prov., Gongshan Co., Dulongjiang Township. 27°40'43"N, 98°16'13"E, elev. 1155 m, 3 April 2012, Zhi-Zhong Yang leg. ***Paratype*** • 1♀ (SWUC-T-LY-28-02) with same data as for holotype.

**Figure 1.**
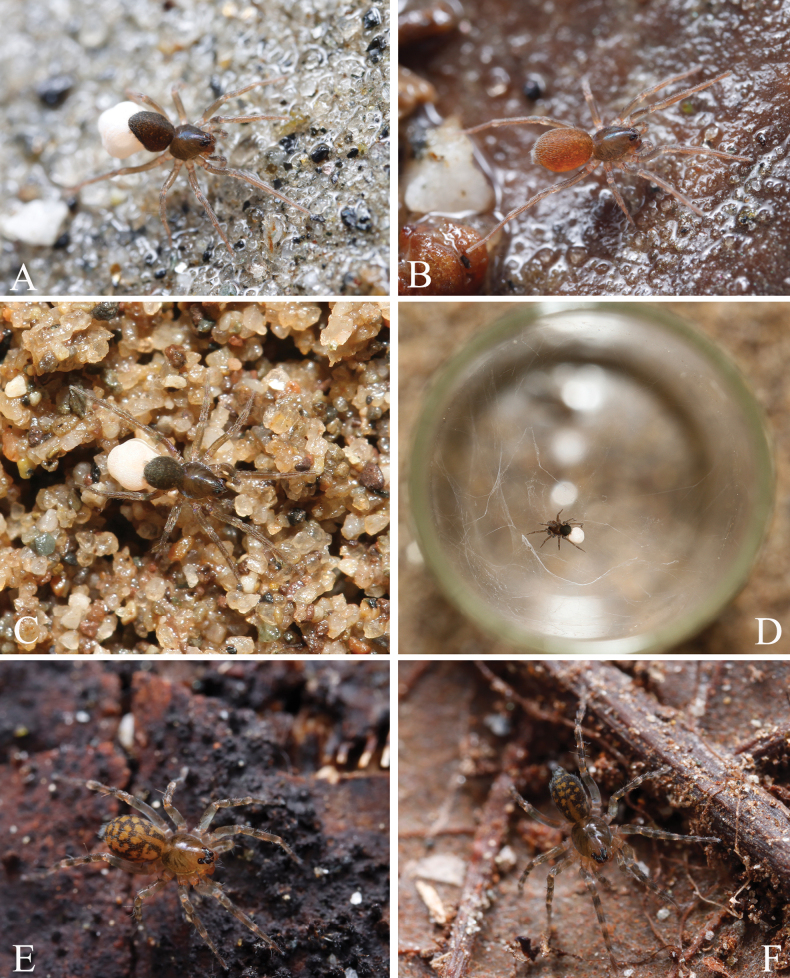
Photos of living *Zoica* spp. A, B. *Zoica medogensis* sp. nov.; C, D. *Zoica thailandica* sp. nov.; E, F. *Zoica oculata* Buchar, 1997 (female, A, B, E, F. Photographed by Qian-Le Lu; C, D. Photographed by Lu-Yu Wang).

##### Etymology.

The specific name comes from the word “Dulong”, the name of one of the Chinese ethnic minorities that live adjacent to the area inhabited by the new species; noun in apposition.

##### Diagnosis.

**Figure 2.**
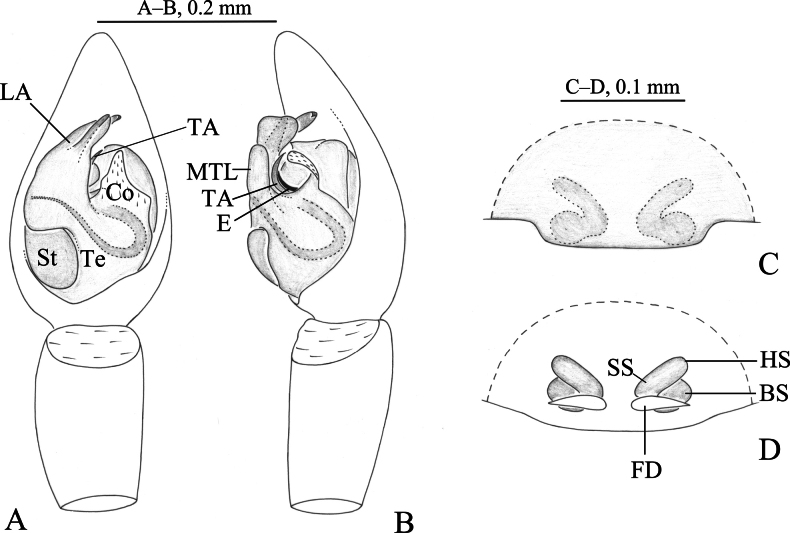
*Zoica dulong* sp. nov. A, B. Holotype male; C, D. Paratype female. A. Left male palp, ventral view; B. Same, prolateral view; C. Epigyne, ventral view; D. Vulva, dorsal view. Abbreviations: BS–base of the spermatheca; Co–conductor; E–embolus; FD–fertilization duct; HS–head of spermatheca; LA–lateral apophysis; MTL–median tegular lobe; SS–stalk of spermatheca; St-subtegulum; TA–terminal apophysis; Te–tegulum.

**Figure 3.**
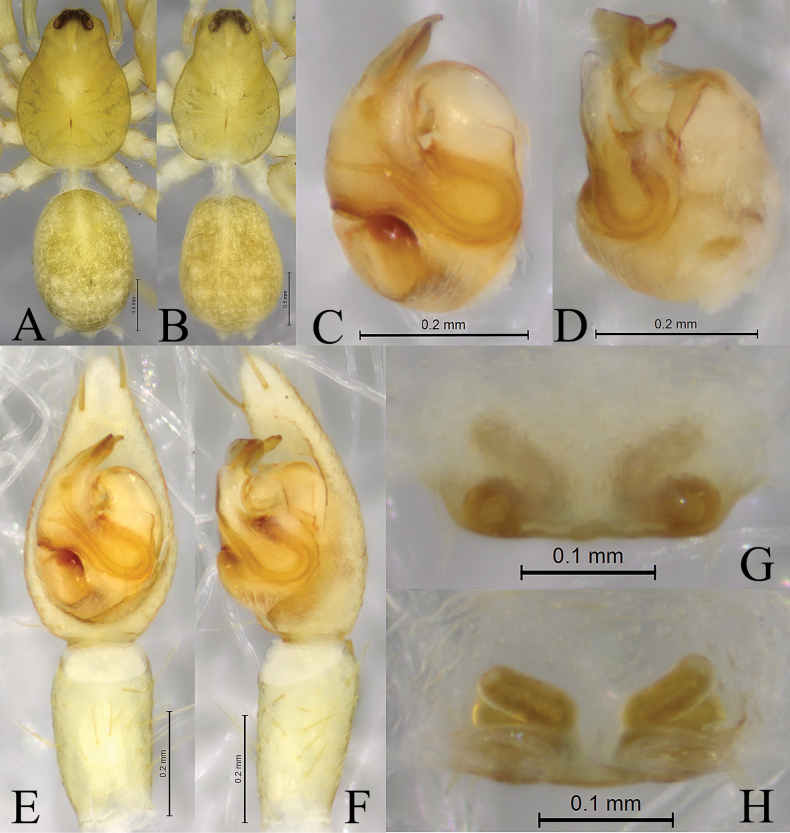
*Zoica dulong* sp. nov. A, C–F. Holotype male; B, G, H. Paratype female. A. Male habitus, dorsal view; B. Female habitus, dorsal view; C. Right male palp (overturn), bulb, ventral view; D. Same, prolateral view; E. Left male palp, ventral view; F. Same, retrolateral view; G. Epigyne, ventral view; H. Vulva, dorsal view.

The new species resembles *Z. puellula* (Simon, 1898) (Sankaran and Sebastian 2017: 148, figs 3A–G) in having bifurcated lateral apophysis and short-widen posterior lip of epigyne (Figs 2, 3C–H), but can be differentiated by the ventral arm of lateral apophysis somewhat rectangular in retrolateral view (Figs 2A, 3C, E) (vs somewhat triangular); cylindrical heads of spermathecae (Figs 2D, 3H) (vs somewhat circular).

##### Description.

**Male** holotype (Fig. 3A) total length 2.90. Carapace 1.56 long, 1.12 wide; opisthosoma 1.39 long, 0.98 wide. Carapace yellow brown. Fovea longitudinal, radial furrow indistinct. Eye region black. Eye sizes and interdistances: AME 0.05, ALE 0.05, PME 0.12, PLE 0.11; AME–AME 0.03, AME–ALE 0.02, PME–PME 0.02, PME–PLE 0.03. Clypeus height 0.04. Chelicerae pale yellow, with 3 promarginal and 2 retromarginal teeth. Labium and endites light yellow. Sternum light yellow, shield-shaped, with sparse yellow setae. Legs light yellow. Leg measurements: I 3.57 (1.07, 1.19, 0.72, 0.59); II 3.39 (0.91, 1.13, 0.73, 0.62); III 3.49 (0.96, 1.19, 0.74, 0.60); IV 4.79 (1.25, 1.61, 1.13, 0.80). Leg formula: 4132. Opisthosoma oval. Dorsum yellow brown, with distinct scutum and sparse brown setae. Venter yellowish brown.

***Palp*** (Figs 2A, B, 3C–F). Subtegulum located on anterior-lateral side of the bulb. Lateral apophysis with broad base and 2 arms: dorsal one thin and long, with a hook-shaped tip; ventral arm dilated with porcatus. Embolus slender, in groove of terminal apophysis. Conductor membranous, with broad base and blunt tip. Median tegular lobe oval, membranous.

**Female** paratype (Fig. 3B) total length 3.03. Carapace 1.46 long, 1.05 wide; opisthosoma 1.33 long, 1.00 wide. Eye sizes and interdistances: AME 0.04, ALE 0.05, PME 0.12, PLE 0.08; AME–AME 0.02, AME–ALE 0.02, PME–PME 0.05, PME–PLE 0.06. Clypeus height 0.04. Legs light yellow. Leg measurements: I 3.55 (0.99, 1.20, 0.75, 0.61); II 3.30 (0.98, 1.10, 0.70, 0.52); III 3.12 (0.92, 0.98, 0.68, 0.54); IV 4.56 (1.31, 1.52, 1.05, 0.68). Leg formula: 4123.

***Epigyne*** (Figs 2C, D, 3G, H). Copulatory openings located posteriorly, inconspicuous. Heads of spermathecae rod-shaped, base of the spermathecae short and strong, subspherical. Fertilization ducts crescent-shaped.

##### Habitat.

Living in the leaf litter.

##### Distribution.

Known only from the type locality, Yunnan, China (Fig. 10).

#### ﻿ Zoica medogensis

Taxon classificationAnimaliaAraneaeLycosidae

Lu, Zhang & Wang sp. nov.

5E134DDC-D98C-5943-9026-BA3423F30AD9

https://zoobank.org/974FF08E-BCBB-4666-BF00-6EB4DCD9E363

**Figure 4.**
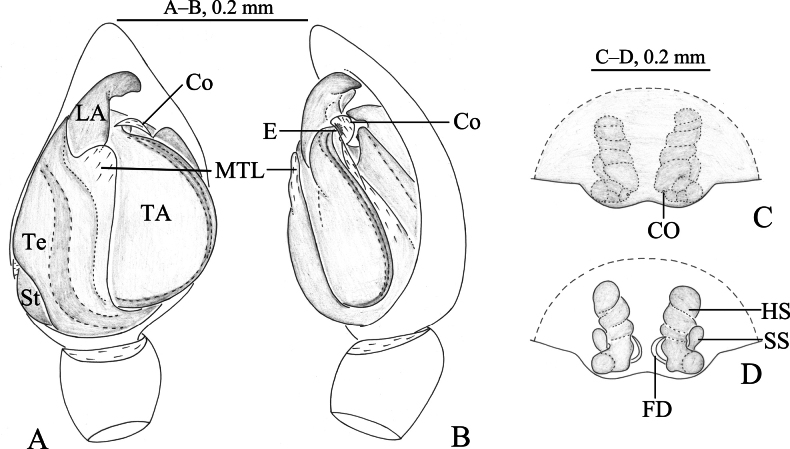
*Zoica medogensis* sp. nov. A, B. Holotype male; C, D. Paratype female. A. Left male palp, ventral view; B. Same, prolateral view; C. Epigyne, ventral view; D. Vulva, dorsal view. Abbreviations: Co–conductor; CO–copulatory opening; E–embolus; FD–fertilization duct; HS–head of spermatheca; LA–lateral apophysis; MTL–median tegular lobe; SS–stalk of spermatheca; St–subtegulum; TA–terminal apophysis; Te–tegulum.

**Figure 5.**
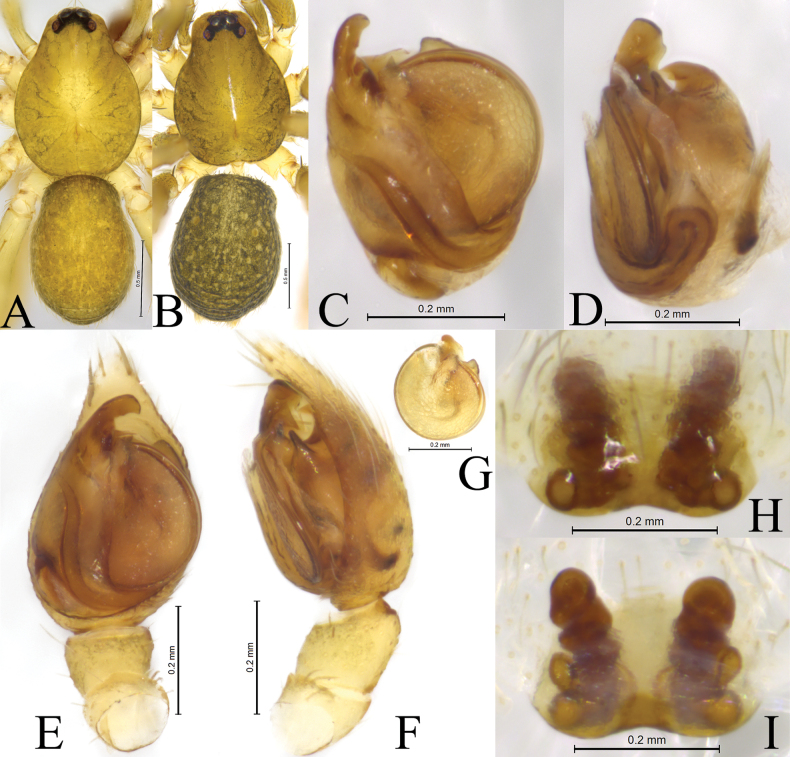
*Zoica medogensis* sp. nov. A, E, F. Holotype male; C, D, G. Paratype male; B, H, I. Paratype female. A. Male habitus, dorsal view; B. Female habitus, dorsal view; C. Left male palp, bulb, ventral view; D. Same, prolateral view; E. Left male palp, ventral view; F. Same, retrolateral view; G. Terminal apophysis and embolus, ventral view; H. Epigyne, ventral view; I. Vulva, dorsal view.

Figs 1A, B , 4 , 5 , 10 墨脱佐卡蛛

##### Type material.

***Holotype*** • ♂ (SWUC-T-LY-29-01), China, Xizang, Medog Co., Yarang Vill., 29°17'48"N, 95°16'48"E, elev. 761 m, 22 May 2019, Lu-Yu Wang, Piao Liu, Tao Yuan and Hui Wang leg. ***Paratypes*** • 4♂ 14♀ (SWUC-T-LY-29-02 to 19) with same data as for holotype • 4♂ 5♀ (SWUC-T-LY-29-20 to 28), Yarang Vill., 30 May 2022, Lu-Yu Wang, Tian-Yu Ren and Bing Tan leg. • 3♀ (SWUC-T-LY-29-29 to 31), Yarang Vill., 28 June 2018, Lu-Yu Wang, Zhi-Sun Wu and Yan-Nan Mu leg. • 1♀ (SWUC-T-LY-29-32), Medog Co., Beibeng Town. 29°14'23"N, 95°10'40"E, elev. 1116 m, 22 May 2019, Lu-Yu Wang, Piao Liu, Tao Yuan and Hui Wang leg. • 1♀ (SWUC-T-LY-29-33), Medog Co., nr Dexing Bridge. 29°19'16"N, 95°17'39"E, elev. 724 m, 23 May 2019, Lu-Yu Wang, Piao Liu, Tao Yuan and Hui Wang leg.

##### Etymology.

The specific name is derived from the type locality: Medog County, Tibet. Adjective.

##### Diagnosis.

*Zoica medogensis* is distinguished from all other congeners by the disciform terminal apophysis (Figs 4A, 5C, E, G) and spirally stalk of spermathecae (Figs 4C, D, 5H, I).

##### Description.

**Male** holotype (Fig. 5A) total length 2.08. Carapace 1.11 long, 0.84 wide; opisthosoma 0.97 long, 0.69 wide. Carapace smooth, yellow brown. Fovea longitudinal, radial furrow indistinct. Eye region black. Eye sizes and interdistances: AME 0.05, ALE 0.05, PME 0.01, PLE 0.07; AME–AME0.01, AME–ALE 0.01, PME–PME0.02, PME–PLE 0.02. Clypeus height 0.04. Chelicerae yellow brown, with three promarginal teeth and two retromarginal teeth. Labium yellowish brown. Endites yellowish brown. Legs yellow brown. Leg measurements: I 2.57 (0.70, 0.86, 0.57, 0.44); II 2.30 (0.65, 0.79, 0.46, 0.40); III 2.09 (0.59, 0.68, 0.49, 0.33); IV 3.13 (0.88, 1.06, 0.78, 0.46). Leg formula: 4123. Opisthosoma oval. Dorsum yellow brown, with sparse brown setae. Venter yellowish brown.

***Palp*** (Figs 4A, B, 5C–F). Subtegulum small, located on anterior-lateral side of the bulb. Lateral apophysis robust, with coracoid-shaped end. Embolus slender and long, in groove of discoid terminal apophysis. Conductor slender, tip slightly expanded, membranous. Median tegular lobe semicircular.

**Female** paratype (SWUC-T-LY-29-02, Fig. 5B) total length 2.39. Carapace 1.19 long, 0.90 wide; opisthosoma 1.15 long, 0.85 wide. Eye sizes and interdistances: AME 0.05, ALE 0.06, PME 0.11, PLE 0.08; AME–AME 0.01, AME–ALE 0.01, PME–PME 0.02, PME–PLE 0.04. Clypeus height 0.03. Leg measurements: I 3.02 (0.87, 1.06, 0.64, 0.45); II 2.62 (0.78, 0.86, 0.55, 0.43, 2.62); III 2.36 (0.67, 0.80, 0.51, 0.38); IV 3.75 (1.03, 1.31, 0.87, 0.54). Leg formula: 4123. Opisthosoma oval. Dorsum yellow brown, with black markings and muscle spots. Ventral yellowish brown.

***Epigyne*** (Figs 4C, D, 5H, I). Copulatory openings small. Stalks of spermathecae strongly twisted. Heads of spermathecae small, suboval. Fertilization duct crescent-shaped.

##### Variation.

Males (*N* = 9) total length 2.07–2.20, females (*N* = 24) total length 2.17–2.41.

##### Habitat.

Living in the leaf litter layer.

##### Distribution.

Known only from the type locality, Xizang, China (Fig. 10).

#### ﻿ Zoica thailandica

Taxon classificationAnimaliaAraneaeLycosidae

Lu, Zhang & Wang sp. nov.

4FD89AAC-EF8A-58C5-9EC0-8E46AF78182D

https://zoobank.org/5D12401-733B-47E5-AF8D-E7EC2FADAFDF

**Figure 6.**
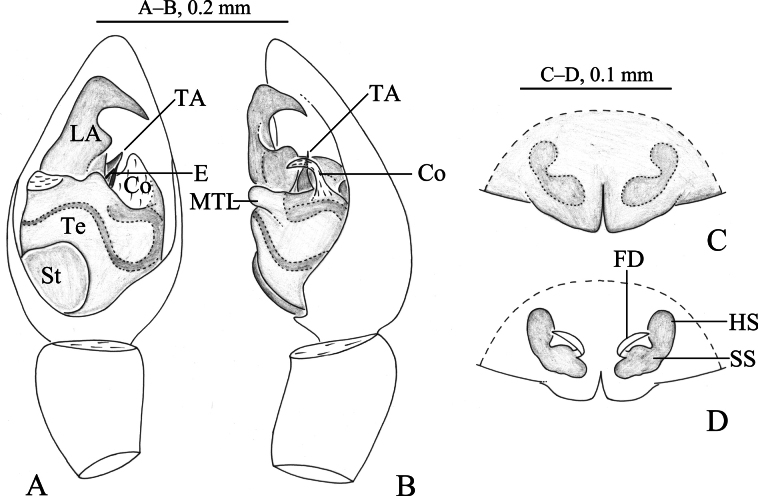
*Zoica thailandica* sp. nov. A, B. Holotype male; C, D. Paratype female. A. Left male palp, ventral view; B. Same, prolateral view; C. Epigyne, ventral view; D. Vulva, dorsal view. For abbreviations, see Fig. 2.

**Figure 7.**
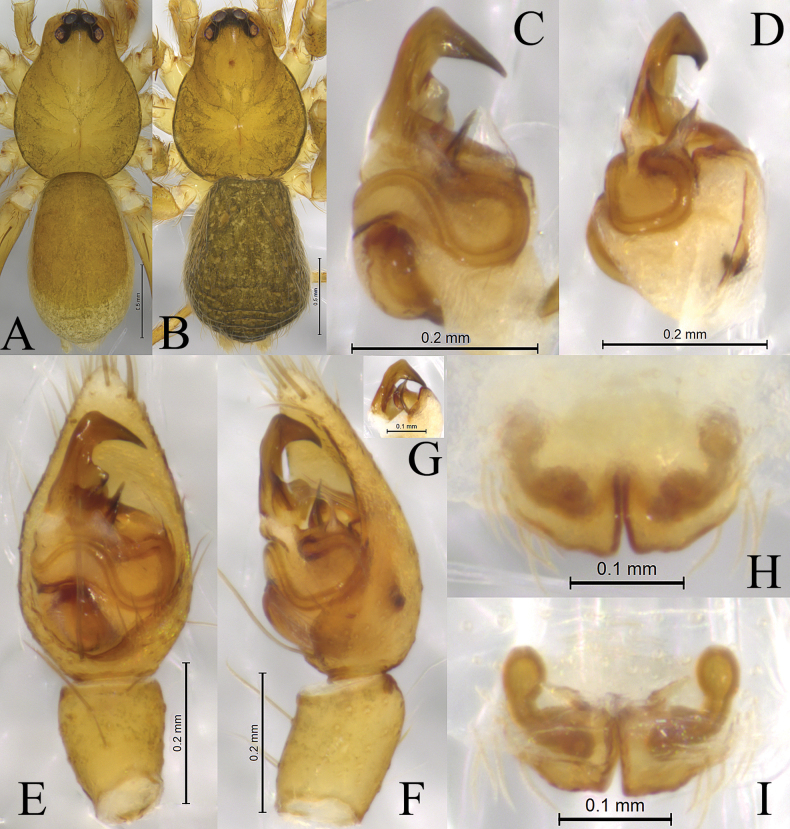
*Zoica thailandica* sp. nov. A, E, F. Holotype male; C, D, G. Paratype male; B, H, I. Paratype female.A. Male habitus, dorsal view; B. Female habitus, dorsal view; C. Left male palp, bulb, ventral view; D. Same, prolateral view; E. Left male palp, ventral view; F. Same, retrolateral view; G. Terminal part, obliquely retrolateral view; H. Epigyne, ventral view; I. Vulva, dorsal view.

Figs 1C, D , 6 , 7 , 10 泰佐卡蛛

##### Type material.

***Holotype*** • ♂ (SWUC-T-LY-30-01), Thailand, Ratchaburi, Suan Pheung Dist., Botanical Garden, 13°31'31"N, 99°14'30"E, elev. 188 m, 21 November 2018, Lu-Yu Wang, Tian Lu and Zheng Fan leg. (SWUC). ***Paratypes***: • 11♂ 12♀ (SWUC-T-LY-30-02 to 24) with same data as for holotype (SWUC) • 3♂ 13♀ (SWUC-T-LY-30-25 to 40), Uthai Thani, Ban Rai, Huai Kha Khaeng Wildlife Sanctuary, 15°17'4"N, 99°28'43"E, elev. 230 m, 25 November 2018, Lu-Yu Wang, Yan-Nan Mu and Tian Lu leg.

##### Etymology.

The species is named after the country where the type locality is situated. Adjective.

##### Diagnosis.

The new species resembles *Z. carolinensis* Framenau, Berry & Beatty, 2009 (Framenau et al. 2009: 228, figs 5–8) in having hook-shaped lateral apophysis and a posterior lip of the epigyne (Figs 6, 7C–I), but can be differentiated from the latter by the long lateral apophysis, almost half length of the bulb (Figs 6A, B, 7C–G; vs short), the posterior lip of the epigyne divided distinctly (Figs 6C, D, 7H, I; vs fused).

##### Description.

**Male** holotype (Fig. 7A) total length 2.26. Carapace 1.16 long, 0.87 wide; opisthosoma 1.17 long, 0.73 wide. Carapace smooth, yellow brown. Fovea longitudinal, radial furrow indistinct. Eye region black. Eye sizes and interdistances: AME 0.04, ALE 0.05, PME 0.11, PLE 0.07; AME–AME 0.02, AME–ALE 0.02, PME–PME 0.03, PME–PLE 0.04. Clypeus height 0.04. Chelicerae yellow brown, with three promarginal teeth and two retromarginal teeth. Labium yellowish brown, longer than wide. Endites yellowish brown, longer than wide. Sternum yellowish brown, with yellow brown setae. Legs yellow brown. Leg measurements: I 2.97 (0.86, 0.98, 0.64, 0.49); II 2.67 (0.79, 0.85, 0.57, 0.46); III 2.17 (0.64, 0.61, 0.52, 0.40); IV 3.56 (0.96, 1.14, 0.90, 0.56). Leg formula: 4123. Opisthosoma oval, with dorsal scutum. Dorsum yellow brown. Venter yellowish brown.

***Palp*** (Figs 6A, B, 7C–G). Subtegulum located on lateral side of the bulb. Lateral apophysis robust, F-shaped, with hook-shaped tip and medial process. Embolus slender, in groove of terminal apophysis (basally swollen, apically pointed). Conductor short, base broad, apex tapering, membranous. Median tegular lobe finger-shaped, membranous.

**Female** paratype (SWUC-T-LY-30-02, Fig. 7B) total length 2.18. Carapace 1.13 long, 0.87 wide; opisthosoma 1.10 long, 0.83 wide. Eye sizes and interdistances: AME 0.03, ALE 0.04, PME 0.10, PLE 0.07; AME–AME 0.01, AME–ALE 0.02, PME–PME 0.04, PME–PLE 0.04. Clypeus height 0.04. Legs yellow brown. Leg measurements: I 2.72 (0.79, 0.95, 0.55, 0.43); II 2.38 (0.74, 0.74, 0.49, 0.41); III 2.16 (0.64, 0.61, 0.52, 0.39); IV 3.35 (0.93, 1.14, 0.77, 0.51). Leg formula: 4123. Opisthosoma yellow brown dorsally. Ventral yellow brown.

***Epigyne*** (Figs 6C, D, 7H, I). Copulatory openings located posteriorly, inconspicuous. Heads of spermathecae spherical. Stalks of spermathecae robust, pear-shaped. Fertilization duct crescent-shaped. The posterior lip of the epigyne divided distinctly.

##### Variation.

Males (*N* = 15) total length 2.15–2.43, females (*N* = 25) total length 2.16–2.73.

##### Habitat.

Living on the sandy ground by the stream.

##### Distribution.

Thailand (Ratchaburi and Uthai Thani) (Fig. 10).

#### ﻿ Zoica oculata

Taxon classificationAnimaliaAraneaeLycosidae

Buchar, 1997

7C20DDC2-7CF8-53BB-B58F-91B9459C85E2

**Figure 8.**
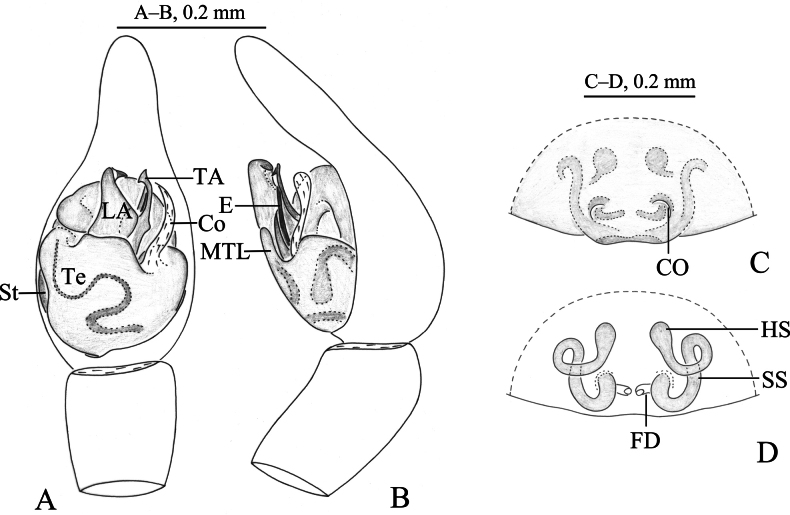
*Zoica oculata* Buchar, 1997. A, B. Male; C, D. Female. A. Left male palp, ventral view; B. Same, prolateral view; C. Epigyne, ventral view; D. Vulva, dorsal view. Abbreviations: Co–conductor; CO–copulatory opening; E–embolus; FD–fertilization duct; HS–head of spermatheca; LA–lateral apophysis; MTL–median tegular lobe; SS–stalk of spermatheca; St–subtegulum; TA–terminal apophysis; Te–tegulum.

**Figure 9.**
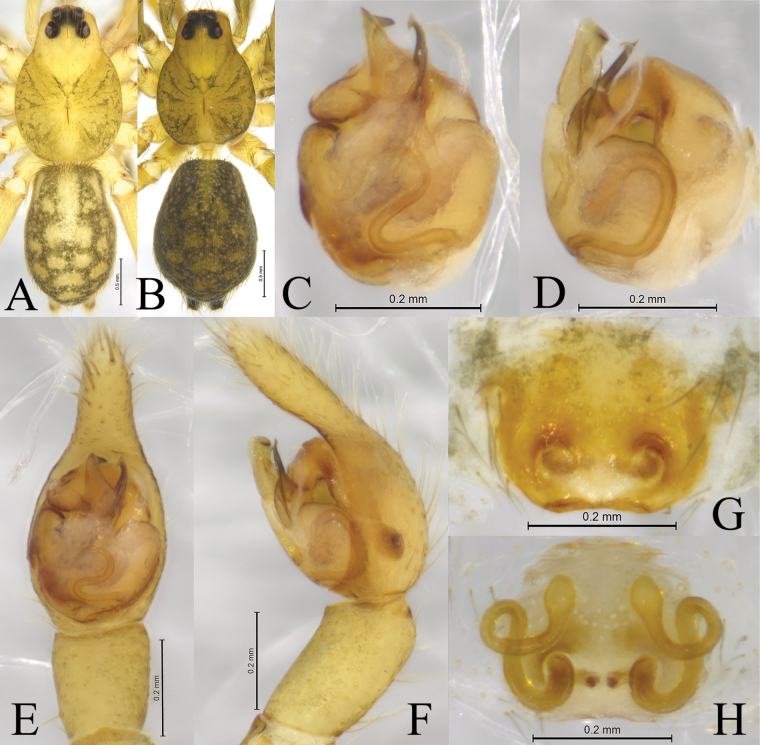
*Zoica oculata* Buchar, 1997. A, C–F. Male; B, G, H. Female. A. Male habitus, dorsal view; B. Female habitus, dorsal view; C. Left male palp, bulb, ventral view; D. Same, prolateral view; E. Left male palp, ventral view; F. Same, retrolateral view; G. Epigyne, ventral view; H. Vulva, dorsal view.

**Figure 10.**
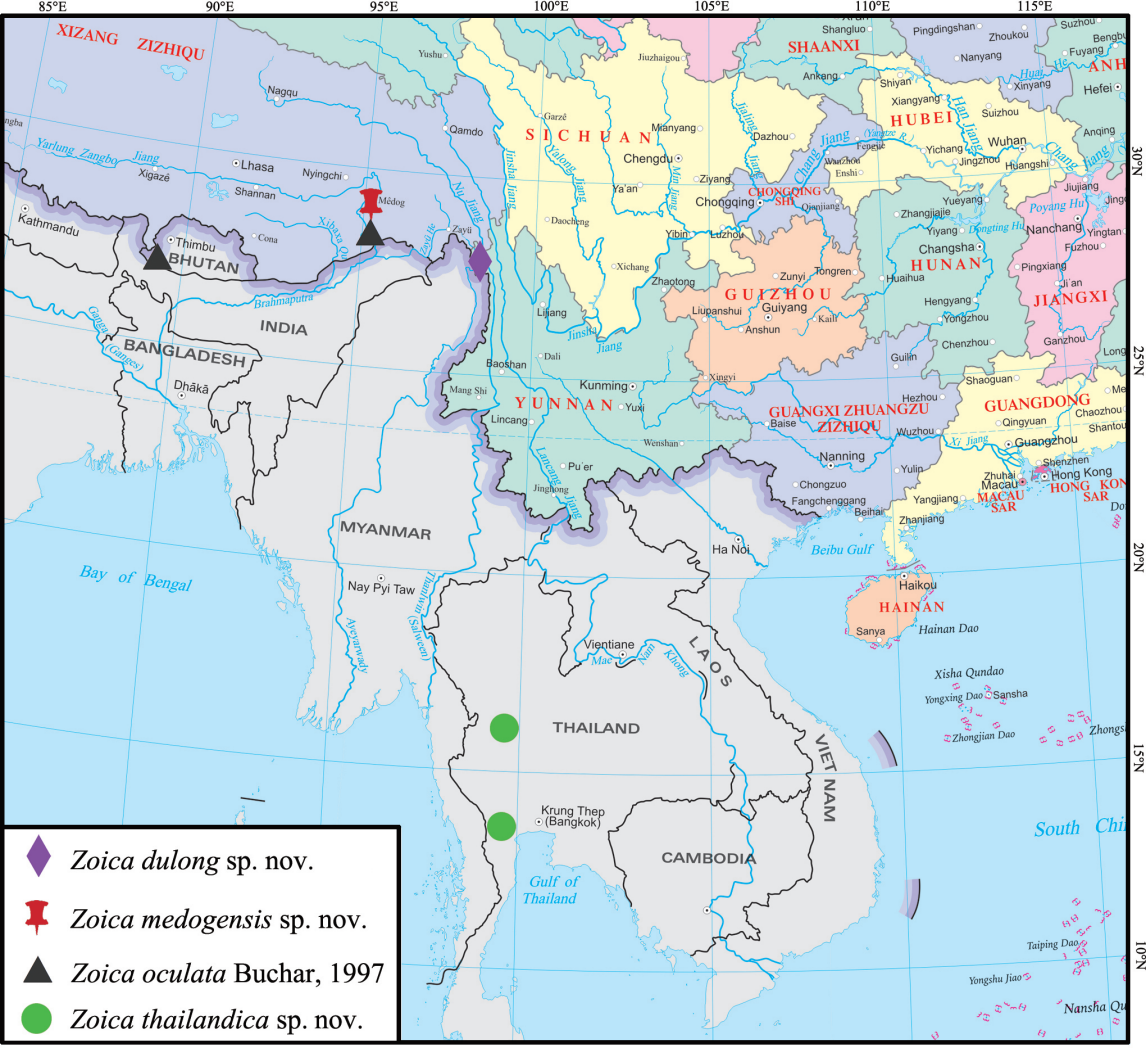
Distribution records of four *Zoica* species treated in this study.

Figs 1E, F , 8 , 9 , 10 眼佐卡蛛

Zoica oculata Buchar, 1997: 7, figs 1–4 (♀).

##### Material.

• 3♂ 4♀ (XZMT-19-39), China, Xizang, Medog Co., Beibeng Town, 29°15'51"N, 95°11'01"E, elev. 894 m, 23 May 2019, Lu-Yu Wang, Piao Liu, Tao Yuan and Hui Wang leg. • 4♀ (XZMT-18-74), Beibeng Town, 28 June 2018, Lu-Yu Wang, Zhi-Sun Wu and Yan-Nan Mu leg. • 1♀ (XZMT-18-77), Medog Co., near Dexing Bridge, 29°19'17"N, 95°17'40"E, elev. 724 m, 29 June 2018, Lu-Yu Wang, Zhi-Sun Wu and Yan-Nan Mu leg. • 2♀ (XZMT-18-78), Medog Co., Dexing Township, Guoguotang Daguiwan, 29°19'34"N, 95°16'22"E, elev. 1025 m, 29 June 2018, Lu-Yu Wang, Zhi-Sun Wu and Yan-Nan Mu leg.

##### Diagnosis.

*Zoica oculata* can be distinguished from all other congeners by the large body (2.77–4.26), long and hook-shaped terminal apophysis, the narrow and long conductor, the long twining stalk of spermathecae (Figs 8, 9C–H), and the block-shaped markings in opisthosoma (Fig. 1E, F, Buchar 1997: fig. 1).

##### Description.

**Male** (Fig. 9A) total length 3.26. Carapace 1.69 long, 1.18 wide; opisthosoma 1.55 long, 1.02 wide. Carapace smooth, yellowish brown. Fovea longitudinal, radial furrows indistinct. Eye region black. Eye sizes and interdistances: AME 0.09, ALE 0.06, PME 0.17, PLE 0.13; AME–AME 0.03, AME–ALE 0.02, PME–PME 0.08, PME–PLE 0.10. Clypeus height 0.05. Chelicerae yellow brown, with three promarginal teeth and four retromarginal teeth. Labium yellowish brown, longer than wide. Endites yellowish brown, longer than wide. Sternum yellow brown, shield-shaped, with sparse yellow brown setae. Legs yellowish brown. Leg measurements: I 5.25 (1.41, 1.75, 1.28, 0.81); II 4.56 (1.25, 1.48, 1.14, 0.69); III 4.32 (1.19, 1.31, 1.15, 0.67); IV 6.39 (1.75, 2.07, 1.79, 0.78). Leg formula: 4123. Opisthosoma oval. Dorsum yellowish brown, with gray markings. Ventral yellowish brown.

***Palp*** (Figs 8A, B, 9C–F). Subtegulum located on medio-lateral side of the bulb. Lateral apophysis base robust, apex hook-shaped. Terminal apophysis long, with a robust base and a hook-shaped end. Embolus curved, moderately long. Conductor slender, membranous. Median tegular lobe broad, adjacent to lateral apophysis, originating from median tegular.

**Female** (Fig. 9B) total length 3.15. Carapace 1.47 long, 1.07 wide; opisthosoma 1.53 long, 1.13 wide. Eye sizes and interdistances: AME 0.13, ALE 0.15, PME 0.06, PLE 0.05; AME–AME 0.06, AME–ALE 0.09, PME–PME 0.02, PME–PLE 0.04. Clypeus height 0.04. Leg measurements: I 4.20 (1.18, 1.58, 0.91, 0.53); II 3.68 (1.08, 1.22, 0.82, 0.56); III 3.45 (0.97, 1.10, 0.87, 0.51); IV 5.10 (1.42, 1.64, 1.38, 0.66). Leg formula: 4123. Opisthosoma oval. Dorsum yellow brown, with gray markings.

***Epigyne*** (Figs 7C, D, 8G, H). Epigynal plate glasses-like. Copulatory openings small, arc-shaped. Stalk of spermathecae long, slender and curved. Heads of spermathecae small, suboval. Fertilization ducts hook-shaped.

##### Variation.

Males (*N* = 3) total length 2.77–3.26, females (*N* = 11) total length 3.11–4.26.

##### Habitat.

Leaf litter, rice field and scrub-grassland.

##### Distribution.

China (Xizang), Bhutan (Fig. 10).

## ﻿Discussion

Lehtinen and Hippa (1979) mentioned that the genus *Zoica* is non-web-building; however, we observed web construction when rearing them in glass tubes (Fig. 1D). Despite this, we did not notice any webs during collection, possibly because they were too minuscule to be detected. Although we observed web-spinning behavior in *Z. thailandica*, the spinneret morphology of this species is morphologically comparable to that of non-web-spinning wolf spiders (Lycosidae), lacking specialized adaptations for web construction. We speculate that *Zoica* might spin small sheet webs within their nests.

## Supplementary Material

XML Treatment for Zoica

XML Treatment for Zoica dulong

XML Treatment for Zoica medogensis

XML Treatment for Zoica thailandica

XML Treatment for Zoica oculata

## ﻿Additional information

### Use of AI

No use of AI was reported.

### Author ORCIDs

Jin-Zhen Lu https://orcid.org/0009-0006-1130-2954

Yuri M. Marusik https://orcid.org/0000-0002-4499-5148

Zhi-Sheng Zhang https://orcid.org/0000-0002-9304-1789

Lu-Yu Wang https://orcid.org/0000-0002-5250-3473

### Data availability

All of the data that support the findings of this study are available in the main text.
